# Subjective age and positive psychiatry: Identifying the positive characteristics associated with successful aging

**DOI:** 10.1192/j.eurpsy.2022.462

**Published:** 2022-09-01

**Authors:** J. Lam, A. Aftab, E. Lee, D. Jeste

**Affiliations:** 1 Warren Alpert Medical School of Brown University, Psychiatry, Providence, United States of America; 2 Case Western Reserve University, Department Of Psychiatry, Cleveland, United States of America; 3 University of California San Diego, Department Of Psychiatry, La Jolla CA, United States of America; 4 University of California San Diego, Department Of Psychiatry, La Jolla, United States of America

**Keywords:** optimism, social support, resilience, Geriatric Psychiatry

## Abstract

**Introduction:**

For older adults, feeling subjectively younger is associated with improvements in cognition, subjective well-being and depressive symptoms. Positive psychiatry is the field that focuses on patient strengths and the promotion of positive outcomes, rather than just mitigation of illness. Younger subjective age may be a useful measure of successful aging, but little is known about how subjective age is associated with positive psychosocial characteristics.

**Objectives:**

Our objective is to characterize how subjective age is related validated positive psychosocial measures, with the goal of better understanding the determinants of successful aging.

**Methods:**

The Successful Aging Evaluation (SAGE) longitudinal study recruited over 1,300 community-dwelling residents of San Diego County, CA, from age 21 to over 100. A single-item question asked “How old/young do you feel?” We used spearman correlations to assess the relationship between subjective age and validated positive psychosocial scales such as the Self-Rated Successful Aging, Life Orientation Test, Personal Mastery Scale, Connor-Davidson Resilience Scale, Satisfaction with Life Scale, Adult Hope Scale, and Social Support Index.

**Results:**

Mean chronological age was 65.5, and mean subjective age was 53.6. Mean age discrepancy was 11.5 years. Younger subjective age was positively associated with most of the positive psychosocial characteristics measured, including self-rated successful aging, optimism, personal mastery, resilience, curiosity, hope, and social support.

**Conclusions:**

There is a growing movement within psychiatry to understand the positive characteristics that lead to successful aging. This is one of the first studies demonstrating younger age identities are associated with positive psychosocial characteristics and successful aging.

**Disclosure:**

No significant relationships.

